# A genome-wide association study identifies single nucleotide polymorphisms associated with time-to-metastasis in colorectal cancer

**DOI:** 10.1186/s12885-019-5346-5

**Published:** 2019-02-09

**Authors:** Michelle E. Penney, Patrick S. Parfrey, Sevtap Savas, Yildiz E. Yilmaz

**Affiliations:** 1 Discipline of Genetics, Faculty of Medicine, Memorial University of Newfoundland, St. John’s, Canada; 2 Discipline of Medicine, Faculty of Medicine, Memorial University of Newfoundland, St. John’s, Canada; 3 Discipline of Oncology, Faculty of Medicine, Memorial University of Newfoundland, St. John’s, Canada; 4 Department of Mathematics and Statistics, Faculty of Science, Memorial University of Newfoundland, St. John’s, Canada

**Keywords:** Colorectal cancer, Genome-wide association study, Mixture cure model, Single nucleotide polymorphisms, Time-to-metastasis

## Abstract

**Background:**

Differentiating between cancer patients who will experience metastasis within a short time and who will be long-term survivors without metastasis is a critical aim in healthcare. The microsatellite instability (MSI)-high tumor phenotype is such a differentiator in colorectal cancer, as patients with these tumors are unlikely to experience metastasis. Our aim in this study was to determine if germline genetic variations could further differentiate colorectal cancer patients based on the long-term risk and timing of metastasis.

**Methods:**

The patient cohort consisted of 379 stage I-III Caucasian colorectal cancer patients with microsatellite stable or MSI-low tumors. We performed univariable analysis on 810,622 common single nucleotide polymorphisms (SNPs) under different genetic models. Depending on the long-term metastasis-free survival probability estimates, we applied a mixture cure model, Cox proportional hazards regression model, or log-rank test. For SNPs reaching Bonferroni-corrected significance (*p* < 6.2 × 10^− 8^) having valid genetic models, multivariable analysis adjusting for significant baseline characteristics was conducted.

**Results:**

After adjusting for significant baseline characteristics, specific genotypes of ten polymorphisms were significantly associated with time-to-metastasis. These polymorphisms are three intergenic SNPs, rs5749032 (*p* = 1.28 × 10^− 10^), rs2327990 (*p* = 9.59 × 10^− 10^), rs1145724 (*p* = 3 × 10^− 8^), and seven SNPs within the non-coding sequences of three genes: *FHIT* (*p* = 2.59 × 10^− 9^), *EPHB1* (*p* = 8.23 × 10^− 9^), and *MIR7515* (*p* = 4.87 × 10^− 8^).

**Conclusions:**

Our results suggest novel associations of specific genotypes of SNPs with early metastasis in Caucasian colorectal cancer patients. These associations, once replicated in other patient cohorts, could assist in the development of personalized treatment strategies for colorectal cancer patients.

**Electronic supplementary material:**

The online version of this article (10.1186/s12885-019-5346-5) contains supplementary material, which is available to authorized users.

## Background

A major contributor to the global cancer burden is colorectal cancer. In 2012, this disease was the second most common cancer in males and third in females. In addition, colorectal cancer caused almost 700,000 deaths worldwide in 2012 [1]. A main cause of death in colorectal cancer is metastasis. Several factors are known to have prognostic importance in colorectal cancer, including the tumor stage and MSI status [2]. However, despite the identification of such factors, there is still significant variability in the long-term risk and timing of metastasis that may be further explained by germline genetic variation.

When analyzing metastasis as a clinical outcome in colorectal cancer, it is observed that not all patients in a population-based cohort experience the outcome despite potentially long follow-up times [3, 4]. Consequently, the long-term metastasis-free survival probability estimate for such a cohort plateaus at a non-zero value. This indicates the study cohort consists of a mixture of long-term metastasis-free survivors as well as patients who are susceptible to metastasis within the follow-up time [3, 5–9]. Thus, there might be prognostic factors distinguishing long-term metastasis-free survivors from patients who are susceptible to metastasis as well as factors determining timing of metastasis in the susceptible group [5, 6, 10]. The MSI tumor phenotype is an example of such a prognostic factor, since colorectal cancer patients with stage I-III MSI-H tumors have a favorable prognosis with very low risk of experiencing metastasis [11, 12]. On the other hand, the patient subgroup with stage I-III MSI-L/MSS tumors is a mixture of colorectal cancer patients that are susceptible and non-susceptible to metastasis [3]. Thus, in this study, we focus our efforts on this patient subgroup to explain more of the variability in the long-term risk and timing of metastasis.

Our objective in this study was to identify common SNPs that are associated with the long-term risk and timing of metastasis of Caucasian colorectal cancer in patients with stage I-III MSI-L/MSS tumors using a genome-wide genotype dataset. This study represents the first comprehensive study that aimed to identify the genetic markers that may be associated with the development of metastasis in colorectal cancer.

## Methods

### Patient cohort and genotype data

The patient cohort included in this study is a sub-cohort of the Newfoundland Colorectal Cancer Registry (NFCCR). The NFCCR recruited 750 colorectal cancer patients in Newfoundland and Labrador between 1999 and 2003 [13]. The characteristics of the NFCCR cohort have been described previously [14, 15]. The NFCCR sought consent from participants; if the patient was deceased, consent was sought from a close relative [13]. These patients were followed until April 2010 [16].

Germline DNA extracted from blood was available for 539 patients in NFCCR [17]. These DNA samples were subject to whole-genome SNP genotyping using the Illumina Omni-1 Quad human SNP genotyping platform at an outsourced company (Centrillion Bioscience, USA). Sample quality control steps on the genotype and patient data were previously described by Xu et al. (2015) for another genome-wide survival study [17]. Patients with discordant sex information, accidental duplicates, divergent or non-Caucasian ancestry, and first, second, or third-degree relatives were removed from the sample cohort [17]. There were 505 patients remaining in this quality-controlled data.

In the previous genome-wide survival study [17], stage I-IV colorectal cancer patients were examined to investigate associations between overall and disease-free survival times and genetic polymorphisms with a minor allele frequency (MAF) of at least 5%. The present study differs from the previous study in terms of the outcome of interest examined, MAFs of the genetic variants (to ensure the inclusion of high-effect low-frequency variants), and patients included in the study, as well as the method of analysis and research question.

**Fig. 1:**
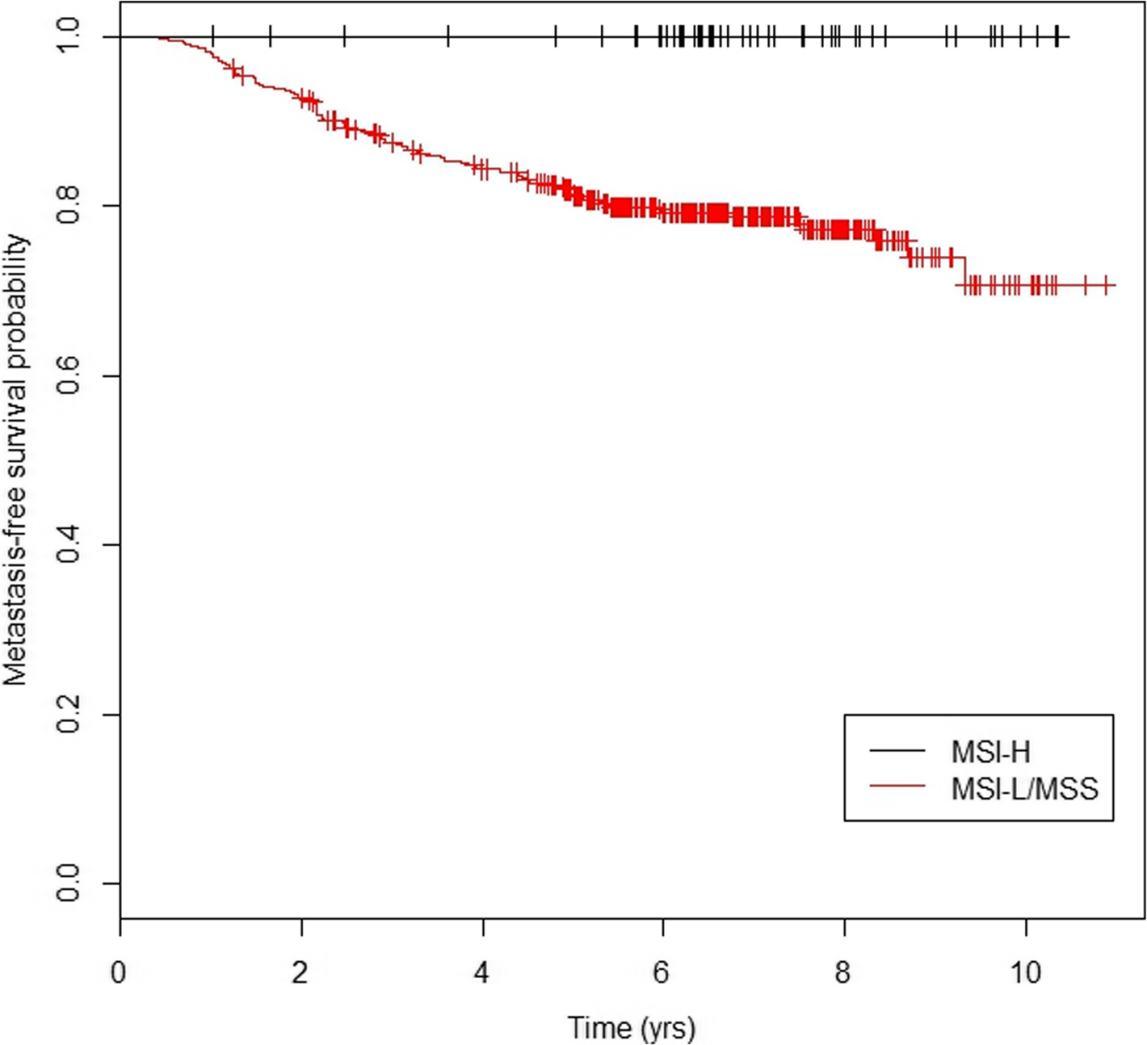
Kaplan-Meier survival functions stratified by microsatellite instability (MSI) status**.** Kaplan-Meier survival functions stratified according to MSI status for the sub-cohort excluding stage IV patients and patients with unknown MSI tumor data (*n* = 431). MSI-H: microsatellite instability high; MSI-L: microsatellite instability low; MSS: microsatellite stable

Further exclusion criteria were applied to the patient data to address the objectives of this study. Only stage I-III patients were considered since patients with stage IV tumors (*n* = 50) already have metastatic cancer. Also, we focus our efforts on the MSI-L/MSS tumor subgroup. This was motivated by the survival pattern observed when stratifying based on MSI status (excluding 20 patients with missing/ unknown MSI tumor status and four patients due to lack of disease recurrence data). In the quality-controlled patient data of stage I-III patients, there are no occurrences of metastasis in patients with MSI-H tumors (Fig. 1). For this reason, 52 patients with MSI-H tumors were excluded. The final study cohort consisted of 379 stage I-III patients with MSI-L/MSS tumors. Of these 379 patients, 21% experienced metastasis. The median follow-up time-to-metastasis was 6.3 years with the longest follow-up time being 10.9 years.

Finally, SNPs whose frequencies deviated from Hardy-Weinberg equilibrium, SNPs with > 5% missing values, and rare SNPs with MAF <1% were excluded, leaving 810,622 SNPs.

### Statistical analysis

The survival outcome of interest throughout the analysis was time-to-metastasis. Patients who did not experience metastasis by the end of the follow-up time were censored at the time of the last follow-up. As seen in Fig. 1, the long-term metastasis-free survival probability estimate for the patient subgroup with MSI-L/MSS tumors plateaus at 0.71 after being followed for just over 9 years. Since there is a plateau at a non-zero probability estimate, such a patient cohort can be properly investigated using the mixture cure model [3, 5, 8, 10, 18–21] which can identify novel genetic markers that are associated with (i) being a long-term survivor without metastasis and (ii) the time-to-metastasis in patients who are susceptible to metastasis after diagnosis. This model can make these determinations separately but simultaneously for each genetic marker. However, when investigating high-dimensional data (such as genome-wide genotype data) using a mixture cure model, it is inevitable that the long-term metastasis-free survival probability estimates for a category of some variables will not plateau at a non-zero probability. For such variables, conventional survival models can be applied.

**Fig. 2:**
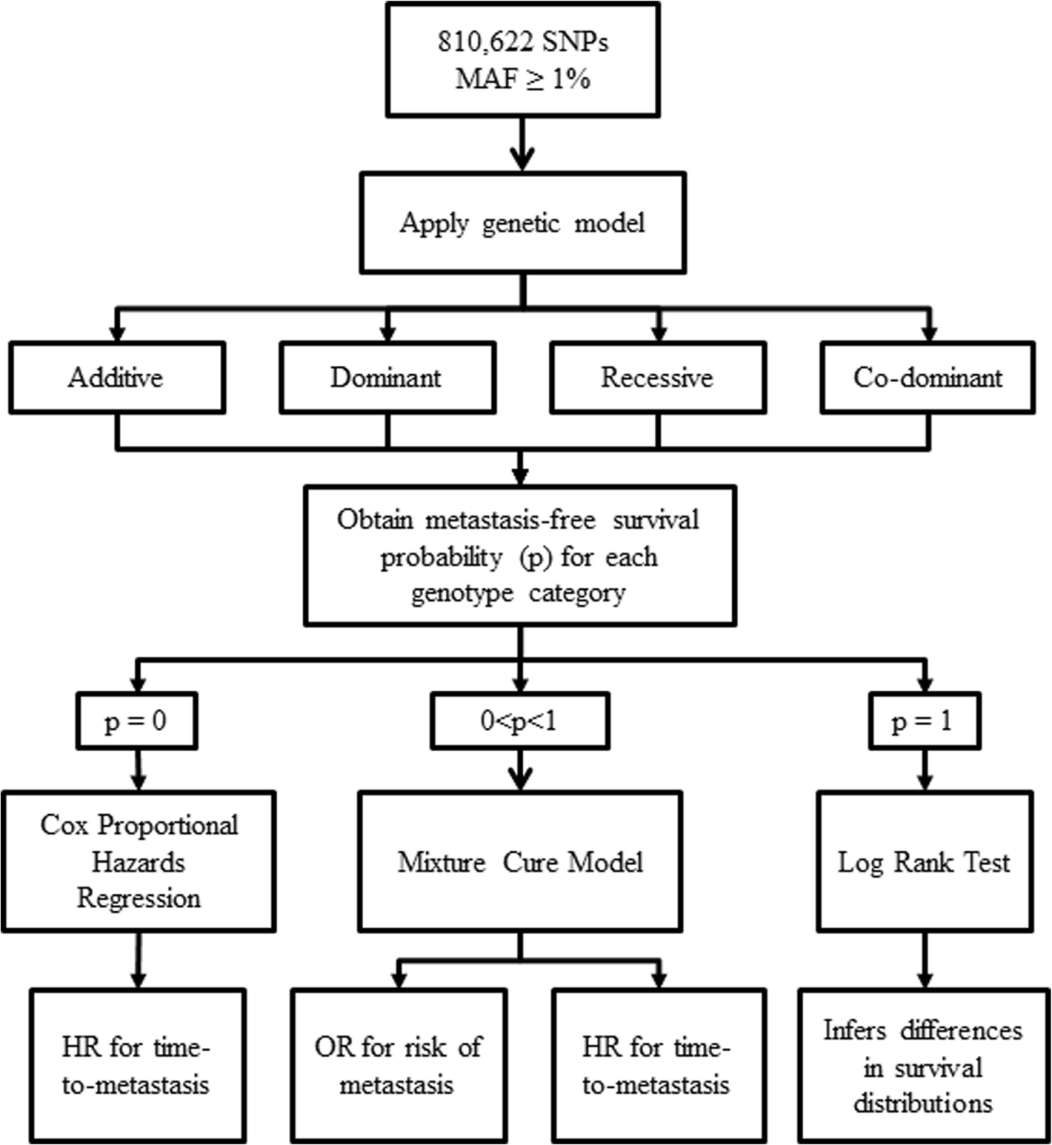
Methods of analysis used in this study. For each SNP, all four genetic models were considered: additive, dominant, recessive, and co-dominant. However, for some SNPs, the number of patients in a genotype category was zero or very small (<2 patients) when the recessive (for 64,809 SNPs) and co-dominant (for 75,912 SNPs) genetic models were applied. As such, these SNPs were not analyzed under these specific genetic models. HR: hazard ratio; MAF: minor allele frequency; OR: odds ratio; p: metastasis-free survival probability

This investigation required a detailed and comprehensive statistical analysis (Fig. 2). Briefly, univariable analysis was performed on genome-wide SNP genotype data under four different genetic models. For each SNP under a given genetic model, in order to determine if the mixture cure model was the appropriate model, we obtained the Kaplan Meier metastasis-free survival probability estimates at the end of the long-term follow-up time for each genotype category. If the long-term metastasis-free survival probability estimates for all genotype categories were between zero and one, the mixture cure model [9] was used. If the long-term metastasis-free survival probability estimate was zero for a genotype category, we applied the Cox proportional hazards regression model. For each significantly associated SNP identified under the Cox proportional hazards regression model, the proportionality assumption was assessed through a score test [22]. If the long-term metastasis-free survival probability estimate was one for a genotype category (i.e. if there is no metastasis within a given subgroup), we applied the log-rank test rather than fitting the mixture cure or Cox proportional hazards model under the corresponding genetic model. SNPs that are associated with the probability of being a long-term metastasis-free survivor and/or the time-to-metastasis in patients who are susceptible to metastasis after diagnosis can be identified using the mixture cure model. For SNPs analyzed using the Cox proportional hazards model, we could test associations between specific genotype categories and time-to-metastasis only. Finally, using the log-rank test, we could determine if there was a significant difference in the survival probability estimates between specified genotype categories.

All four genetic models were considered under the mixture cure model and for the log-rank test. However, only the recessive and co-dominant genetic models were used under the Cox proportional hazards model since there were no SNPs under the additive or dominant genetic models with corresponding genotypes yielding 0% metastasis-free survival estimate.

For each significantly associated SNP, we assessed the fit of the genetic model under which it was identified. Since recessive, dominant, and additive models are nested models of the co-dominant model [23], we compared the results of the identified genetic model to the results of the co-dominant model using maximum likelihood ratios. We performed likelihood ratio tests to assess whether the identified genetic model was the plausible model. Additionally, we compared the coefficient estimates obtained in the identified additive, dominant, or recessive genetic model with the results obtained from the co-dominant model.

Univariable analysis was also performed on the baseline characteristics to identify potential confounding factors to be adjusted for in the multivariable analysis. This analysis was performed using the mixture cure model and the Cox proportional hazards model to select significant baseline characteristics for each model separately. First, all the available baseline characteristics (Table 1) significantly associated with the long-term risk or timing of metastasis for the mixture cure and timing of metastasis for the Cox proportional hazards model in the univariable analysis at a liberal *p*-value threshold of 0.10 were included in the initial multivariable model. Patients with missing or unknown values for the baseline characteristics were excluded from this analysis. As such, we included only patients for which we had all data for the given baseline variable. After fitting this initial model, a backward selection method was applied to obtain the final model using a *p*-value threshold of 0.05. After this step, the significant baseline characteristics in the final multivariable mixture cure model were tumor location, 5-fluorouracil (5-FU) treatment status, and stage (see Additional file 1: Table S1). In the Cox proportional hazards model, the significant baseline characteristics in the final multivariable model were tumor location, stage, and *BRAF* V600E mutation status. In addition, although insignificant in the stepwise selection, 5-FU treatment status was forced into the model (see Additional file 1: Table S1). Of the significant baseline characteristics, only 5-FU treatment status and *BRAF* V600E mutation status had patients with missing or unknown values and, thus, these patients were excluded from both models, resulting in 349 patients. Multivariable analysis adjusting for significant baseline characteristics was performed only on significantly associated SNPs that were identified in their most plausible genetic model.

**Table 1:** Baseline characteristics of the patient cohort (*n* = 379) including metastasis proportion

Variable	Number of patients^a^	% total	Number with metastasis	% metastasis
Sex				
Female	139	36.7%	29	20.9%
Male	240	63.3%	52	21.7%
Age				
≤60	157	41.4%	41	26.1%
60–70	154	40.6%	29	18.8%
> 70	68	17.9%	11	16.2%
Familial risk				
Low	196	51.7%	34	17.3%
Intermediate/High	183	48.3%	47	25.7%
5-FU based treatment				
5-FU treated	214	56.5%	59	27.6%
Other/No chemo	159	42.0%	17	10.7%
Unknown	6	1.6%	5	83.3%
Stage				
I	81	21.4%	8	9.9%
II	158	41.7%	30	19.0%
III	140	36.9%	43	30.7%
Location				
Colon	233	61.5%	41	17.6%
Rectum	146	38.5%	40	27.4%
Histology				
Non-mucinous	343	90.5%	75	21.9%
Mucinous	36	9.5%	6	16.7%
Vascular invasion				
Absence	242	63.9%	45	18.6%
Presence	111	29.3%	30	27.0%
Unknown	26	6.9%	6	23.1%
Lymphatic invasion				
Absence	237	62.5%	44	18.6%
Presence	116	30.6%	31	26.7%
Unknown	26	6.9%	6	23.1%
*BRAF V600E* mutation				
Absence	333	87.9%	72	21.6%
Presence	19	5.0%	8	42.1%
Unknown	27	7.1%	1	3.7%

^a^ Patients with MSI-H tumors and Stage IV patients were excluded. 5-FU: 5-fluorouracil

For the genetic association analyses, a Bonferroni-corrected *p*-value of 6.2 × 10^− 8^ was deemed significant. All statistical analyses were conducted using R v 3.1.3 [24].

### Bioinformatics analysis

To identify any potential reported biological effects of the SNPs reaching genome-wide significance, we conducted a search in Ensembl [25] and RegulomeDB [26] databases. Furthermore, the MAF of each significant SNP was calculated from the patient cohort using PLINK v1.07 [27] to compare their frequencies to the larger CEU population based on 1000 Genomes Project Phase 3 [28].

## Results

The baseline characteristics of the patient cohort can be found in Table 1. The characteristics of the patient cohort considered in this study with genotype data (*n* = 379) were comparable to the larger NFCCR cohort excluding stage IV and MSI-H tumors (*n* = 493) (see Additional file 1: Table S2).

**Fig. 3:**
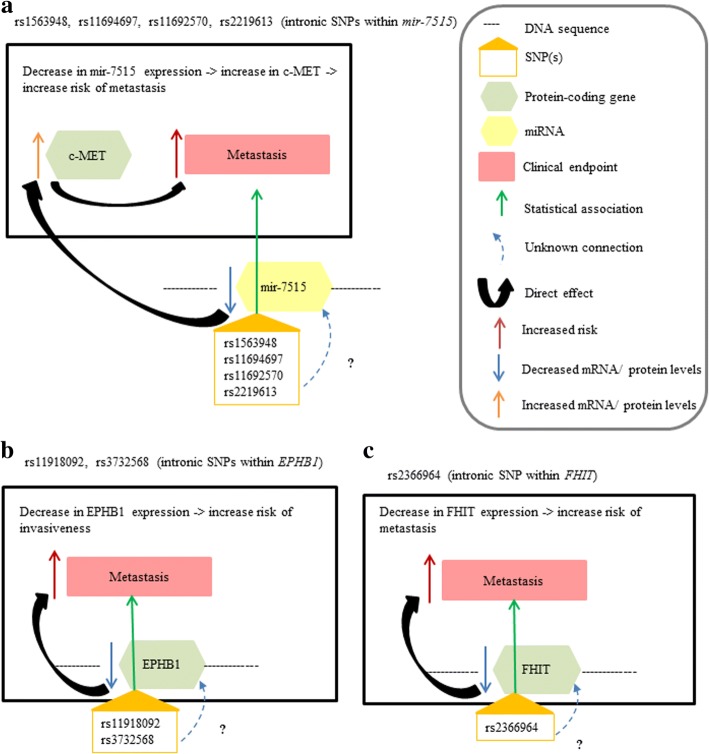
Kaplan-Meier survival function for the most significant SNPs in the multivariable analysis under the (**a**) mixture cure model and (**b**) Cox proportional hazards regression model. n: number of patients in that genotype category; d: number of metastasis in that genotype category. **a** rs5749032 was the only SNP maintaining genome-wide significance after the multivariable analysis using the mixture cure model. In the rs5749032 GG genotype subgroup, the clear plateau at approximately 80% metastasis-free survival probability indicates the existence of a large proportion of long-term metastasis-free survivors. **b** In the rs2327990 TT genotype subgroup, all the patients experienced metastasis within approximately the first two years. Therefore, a standard survival analysis method is appropriate

Using the univariable mixture cure model, we identified specific genotypes of nine SNPs that were significantly associated with time-to-metastasis (see Additional file 1: Table S3 and Additional file 1: Figure S1 and Fig. 3a). These SNPs were identified under the dominant, recessive or co-dominant genetic model and satisfied the test for genetic model validity. Each of the nine significant SNPs was analyzed using a multivariable mixture cure model adjusting for significant baseline characteristics (Table 2). Of these, association of the minor allele homozygous genotype (genotype frequency = 14%) in one SNP remained significant with time-to-metastasis in the multivariable model (GG genotype of rs5749032; HR = 15.86 [95% CI: 6.83–36.83], *p* = 1.28 × 10^− 10^). We also obtained significant associations for some SNPs under the additive model. However, upon checking the validity of the genetic model, we found that the additive genetic model was not plausible for those SNPs. Thus, these results are not reported.

**Table 2:** Results from the multivariable^a^ analysis using the mixture cure model on the significant SNPs identified by the univariable mixture cure model

Genomic location	Genetic model	rs number (genotypes *a* vs. *b*)	Genotype freq.	Metastasis probability			Time-to-metastasis		
				OR	95% CI	*p*-value	HR	95% CI	*p*-value
22:17793969	Recessive	rs5749032 (GG vs. AA + AG)	14%	0.38	0.14–1.07	0.066	15.86	6.83–36.83	1.28 × 10^−10^
17:77361176	Co-Dominant	rs12949587 (CT vs. CC)	20%	0.66	0.32–1.37	0.261	7.56	3.44–16.61	4.63 × 10^−7^
20:15111138	Co-Dominant	rs6110524 (AG vs. GG)	17%	0.95	0.44–2.04	0.887	4.80	2.00–11.53	4.52 × 10^−4^
7:33913404	Recessive	rs3815652 (TT vs. CC + CT)	4%	0.59	0.13–2.65	0.488	12.97	3.26–51.66	2.78 × 10^−4^
14:100691178	Recessive	rs756055 (CC vs. TT + TC)	13%	0.28	0.10–0.82	0.020	7.58	2.53–22.65	2.90 × 10^−4^
14:100730920	Recessive	rs7153665 (AA vs. GG + AG)	13%	0.28	0.10–0.82	0.020	7.58	2.53–22.65	2.90 × 10^−4^
11:100430053	Recessive	rs4754687 (AA vs. CC + CA)	11%	0.51	0.18–1.43	0.201	8.13	2.59–25.53	3.28 × 10^−4^
5:155345221	Dominant	rs2163746 (CT + CC vs. TT)	24%	0.49	0.23–1.07	0.075	9.65	3.67–25.37	4.29 × 10^−6^
5:155361116	Dominant	rs17053011 (TG + TT vs. GG)	24%	0.49	0.23–1.07	0.075	9.65	3.67–25.37	4.29 × 10^−6^

^a^Adjusted for the significant baseline characteristics: tumor location, 5-fluorouracil treatment status, and tumor stage. Each SNP was analyzed separately adjusting for these factors. Patients with missing data were excluded, resulting in the inclusion of 349 stage I-III patients with MSI-L/MSS tumors

Linkage disequilibrium (LD) calculations indicated that rs756055 and rs7153665 as well as rs2163746 and rs17053011 are in complete pairwise LD (r^2^ = 1)

The SNPs listed yielded similar hazard ratio estimates under the univariable (Additional file 1: Table S3) and multivariable analyses. Consequently, all of the SNPs identified in this study could be considered independent prognostic factors for time-to-metastasis in colorectal cancer if the results are replicated using independent cohort data

*Genotype freq.* frequency of genotype *a* calculated from the patient cohort, *OR* odds ratio for metastasis comparing odds of metastasis in subgroup *a* with that in subgroup *b, HR* hazard ratio comparing metastasis rate in subgroup *a* with that in subgroup *b* among those who are susceptible to metastasis, *CI* confidence interval

Univariable analysis under the Cox proportional hazards model identified 25 SNPs that were significantly associated with time-to-metastasis under the recessive and the co-dominant genetic models (see Additional file 1: Table S4 and Fig. 3b). The fitted genetic models were found to be the most plausible genetic model for each SNP and the proportionality assumption was not rejected for any of the significant SNPs. After adjusting for the significant baseline characteristics in the multivariable analysis, specific genotypes of nine SNPs remained significantly associated with time-to-metastasis (Table 3). The most plausible genetic model for each of these nine SNPs was the recessive genetic model. While the confidence intervals are wide due to the small genotype frequencies, the effect sizes are large, which is why the associations reached the conservative genome-wide significance level.

**Table 3:** Genotypes significantly associated with time-to-metastasis after adjusting for significant baseline characteristics identified in the Cox proportional hazards regression model

Genomic location	rs number (genotypes *a* vs. *b*)	Genotype freq.	Univariable			Multivariable^a^		
			HR	95% CI	*p*-value	HR	95% CI	*p*-value
20:16189263	rs2327990 (TT vs. CC + CT)	1.3%	21.97	8.42–57.33	2.74 × 10^− 10^	22.58	8.32–61.31	9.59 × 10^− 10^
3:134513356	rs11918092 (CC vs. AA + AC)	0.5%	216.98	35.64–1321.13	5.32 × 10^− 9^	535.33	63.20–4534.30	8.23 × 10^− 9^
3:134515336	rs3732568 (AA vs. CC + CA)	0.5%	216.98	35.64–1321.13	5.32 × 10^− 9^	535.33	63.20–4534.30	8.23 × 10^− 9^
3:59930672	rs2366964 (CC vs. TT + TC)	0.8%	41.19	11.81–143.66	5.40 × 10^−9^	56.53	14.98–213.26	2.59 × 10^− 9^
2:6769988	rs1563948 (AA vs. GG + GA)	0.8%	34.43	10.35–114.58	7.97 × 10^−9^	33.97	9.57–120.54	4.87 × 10^− 8^
2:6773920	rs11694697 (TT vs. CC + CT)	0.8%	34.43	10.35–114.58	7.97 × 10^−9^	33.97	9.57–120.54	4.87 × 10^− 8^
2:6777992	rs11692570 (TT vs. CC + CT)	0.8%	34.43	10.35–114.58	7.97 × 10^−9^	33.97	9.57–120.54	4.87 × 10^− 8^
2:6779277	rs2219613 (TT vs. CC + CT)	0.8%	34.43	10.35–114.58	7.97 × 10^−9^	33.97	9.57–120.54	4.87 × 10^−8^
6:91187510	rs1145724 (GG vs. AA + AG)	0.8%	30.76	9.27–102.03	2.14 × 10^−8^	36.43	10.21–129.93	3.00 × 10^−8^

^a^Adjusted for tumor location, 5-fluorouracil treatment status, *BRAF* V600E somatic mutation status, and tumor stage. Each SNP was analyzed separately adjusting for these factors. Patients with missing data were excluded, resulting in the inclusion of 349 stage I-III patients with MSI-L/MSS tumors

LD calculations indicated that rs11918092 and rs3732568 are in high pairwise LD (r^2^ = 0.96). In addition, rs1563948, rs11694697, rs11692570, and rs2219613 are all highly linked to each other (0.94 ≤ r^2^ ≤ 1)

The SNPs listed yielded similar risk estimates under the univariable and multivariable analyses. Consequently, all of the SNPs identified in this study could be considered independent prognostic factors for time-to-metastasis in colorectal cancer if the results are replicated using independent cohort data

*Genotype freq.* frequency of genotype *a* calculated from the patient cohort, *HR* hazard ratio comparing metastasis rate in subgroup *a* with that in subgroup *b, CI* confidence interval

Of the SNPs analyzed in this study, there were no associations with the long-term risk of metastasis reaching Bonferroni-corrected significance. However, promising associations were detected and are reported for interested readers (see Additional file 1: Table S5 and Figure S2).

Finally, queries into the Ensembl database [25] revealed that the significantly associated variants were either intronic or intergenic. The intronic SNPs were within the sequences of three different genes: *FHIT, MIR7515*, and *EPBH1*. Furthermore, a search in the RegulomeDB database [26] indicated that the identified SNPs have little reported regulatory significance. Finally, the MAF calculations for this specific cohort are comparable to the Caucasian population based on 1000 Genomes Project Phase 3 [28] (Table 4).

**Table 4:** Variant information for the significant genotypes in the multivariable mixture cure and Cox proportional hazards regression models

Genomic location	rs number (genotype^a^)	MAF^b^	Statistical model^c^	Type of variant (gene)^d^	DNA binding evidence^e^
22:17793969	rs5749032 (GG)	40%	Mixture cure	Intergenic	ND
20:16189263	rs2327990 (TT)	11%	Cox proportional hazards	Intergenic	Less likely to affect binding
3:134513356	rs11918092 (CC)	8%	Cox proportional hazards	Intronic (*EPHB1*)	Minimal binding evidence
3:134515336	rs3732568 (AA)	8%	Cox proportional hazards	Intronic (*EPHB1*)	Minimal binding evidence
3:59930672	rs2366964 (CC)	8%	Cox proportional hazards	Intronic (*FHIT*)	ND
2:6769988	rs1563948 (AA)	11%	Cox proportional hazards	Intronic (*MIR7515*)	Minimal binding evidence
2:6773920	rs11694697 (TT)	11%	Cox proportional hazards	Intronic (*MIR7515*)	ND
2:6777992	rs11692570 (TT)	11%	Cox proportional hazards	Intronic (*MIR7515*)	Minimal binding evidence
2:6779277	rs2219613 (TT)	11%	Cox proportional hazards	Intronic (*MIR7515*)	Minimal binding evidence
6:91187510	rs1145724 (GG)	9%	Cox proportional hazards	Intergenic	Minimal binding evidence

^a^ Risk increasing/decreasing genotype, ^b^ MAF calculated from patient cohort analyzed. Values comparable to CEU population based on 1000 Genomes Project Phase 3 ^28^ data obtained through the Ensembl database (http://grch37.ensembl.org/), ^c^ Statistical model identifying the association, ^d^ based on Ensembl database [25], ^e^ based on RegulomeDB database [26]. ND: no data

## Discussion

Distant metastasis is the most lethal event in colorectal cancer progression. Despite significant advances in treatment options, the 5-year survival rate for metastatic colorectal cancer patients is only 13.5% in the US [29]. Tumor MSI status is an important prognostic indicator in colorectal cancer, as patients with MSI-H tumors rarely experience metastasis [11, 12, 30, 31]. Identifying additional biomarkers that can distinguish between patients who will experience metastasis in the short-term and who will not experience metastasis in the long-term has clear clinical implications in the management and treatment of this disease. In this study, using a focused study design as well as applying appropriate and informative methods of analysis, we identified ten genetic polymorphisms significantly associated with time-to-metastasis in stage I-III Caucasian colorectal cancer patients with MSI-L/MSS tumors after adjusting for significant baseline characteristics.

The mixture cure model identified a specific genotype (GG) of one SNP (rs5749032) that was significantly associated with early metastasis after adjusting for significant baseline characteristics (Table 2, HR = 15.86, *p* = 1.28 × 10^− 10^). This was a frequent genotype in the patient cohort (14%). Most patients with this genotype that experienced metastasis did so within the first 2 years post-diagnosis (Fig. 3a). After this time-point, patients with this genotype did not experience metastasis, despite the long-term follow-up for many patients. Essentially, this suggests that if metastasis occurs in patients with this genotype, it is likely to be in a relatively short time after diagnosis. A search in scientific literature and in the RegulomeDB database [26] did not return information about possible biological or regulatory functions of this polymorphism. In addition, according to the Haploreg database [32], there are no known SNPs in high linkage disequilibrium with this SNP. Thus, this polymorphism may have a direct biological effect on time-to-metastasis. According to the UCSC Human genome browser [33], the rs5749032 polymorphism is within an intergenic sequence flanked by two genes: *CECR2* and *CECR3* (see Additional file 1: Figure S3). CECR2 is a transcription factor that is reported to be involved in chromatin remodeling [34] and may have an additional role in DNA damage response [35]. On the other hand, *CECR3* is a non-coding RNA, according to the Gene Entrez database [36]. Presently, there are no reported relationships between these two genes and cancer. Finally, it is important to note that this association would not have been detected using the Cox proportional hazards model since the proportional hazards assumption was not satisfied (i.e. the survival curves cross; Fig. 3a) and there is a large proportion of long-term metastasis-free survivors (i.e. stable plateau at non-zero metastasis-free survival probability; Fig. 3a). We verified this by fitting a Cox proportional hazards model to this SNP. Under neither the univariable (HR = 1.03 [95% CI: 0.54–1.94], *p* = 0.93) nor the multivariable (HR = 1.09 [95% CI: 0.57–2.10], *p* = 0.80) Cox proportional hazards analysis was there a significant association. Overall, this SNP is a novel candidate biomarker deserving further investigations, particularly replicating its association and examining its potential biological link to metastasis.

For the SNPs with genotype categories showing 0% metastasis-free survival probability, the Cox proportional hazards model identified nine SNPs significantly associated with time-to-metastasis after adjusting for significant baseline characteristics (Table 3, and see Additional file 1: Table S4). Each of the patients with the risk increasing minor allele homozygous genotype of the nine significant SNPs experienced metastasis in a short time after diagnosis (Additional file 1: Figure S4). The most significant SNP, rs2327990, is an intergenic variant (Table 4). While there are no published reports about this SNP, according to the RegulomeDB database [26] there is some evidence that rs2327990 may affect the binding of transcription factors USF1 and USF2. The consequence of this potential regulatory function with regards to metastasis in colorectal cancer has yet to be investigated. A query in the UCSC Human Genome Browser [33] showed this variant is located between a processed pseudogene, *PPIAP17*, and a protein coding gene, *KIF16B*. KIF16B is a kinesin-like protein that may be involved in intracellular trafficking [37]. While the function of *PPIAP17* is not known, there is a protein coding gene further upstream: *MACROD2*. This gene is quite interesting because one study examining 352 colorectal cancer patients identified *MACROD2* as the gene with the most prevalent and recurrent chromosomal breakpoints in colorectal tumors (41%) [38]. According to the Gene Entrez database [36], this gene encodes a deacetylase that removes ADP ribose from modified proteins. As also discussed by van den Broek et al. (2015) [33], one of the target proteins of MACROD2 is GSK3β: active MACROD2 removes the mono-ADP-ribosyl units resulting in an increase in active GSK3β [39]. Interestingly, GSK3β is a regulator of the Wnt signaling pathway [40, 41] and connections between upregulated Wnt signaling and distant metastasis in colorectal cancer have been identified [42, 43]. Thus, when there is a reduction in active MACROD2 levels, this may lead to decreased GSK3β function, which in turn could lead to increased Wnt signaling and, accordingly, an increased risk of metastasis (see Additional file 1: Figure S3). Therefore, evaluating the presence of a link between rs2327990 and MACROD2 expression levels and metastatic potential may prove to be valuable.

**Fig. 4:**
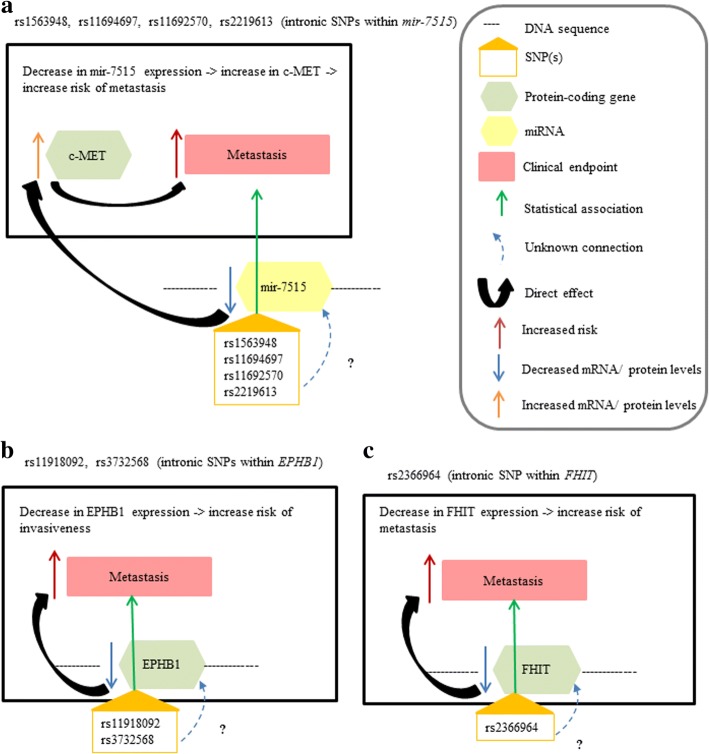
Known and hypothesized relationships between the identified SNPs, genes they are located in, and the risk of metastasis

It is important to note that, although the quality control steps excluded rare SNPs (MAF < 1%), when the recessive and co-dominant genetic models were applied to the raw genotype data, we obtained genotype frequencies that are rare in the patient cohort. This is because these genetic models analyze the minor allele homozygous genotypes as one independent category. As a result, for the remaining eight significant SNPs reported from the Cox proportional hazards model, the genotype frequencies were less than 1% (Table 3). Consequently, although the associations were significant (possibly due to a high effect size [44]), the results may not be generalized to the population. The results require replication in independent cohorts. These SNPs were either intergenic (*n* = 1; rs1145724; see Additional file 1: Figure S3) or located within intronic sequences of three genes (*n* = 7), including four linked SNPs in *MIR7515*, two linked SNPs in *EPHB1*, and one SNP in *FHIT* (Fig. 4), according to the UCSC Human Genome Browser [33]. There are no known functional consequences reported for these SNPs (Table 4) and the potential biological effects of these SNPs on these genes or metastasis in colorectal cancer are not presently known. However, the results of our study combined with previously published findings suggest that there may be potential relationships between these genes and metastasis in colorectal cancer. For example, low levels of FHIT [45, 46] and increased levels of a target of *MIR7515*, c-MET [47], have been linked to increased risk of metastasis of colorectal tumors [48, 49]. In addition, a reduced level of EPHB1 in colorectal cancer cells was associated with increased invasive potential in one study [50].

This is one of the first large-scale association studies that examined clinical outcomes in colorectal cancer. Two other studies published previously investigated the prognostic value of genome-wide genetic polymorphisms on colorectal cancer patient outcomes. As explained in the Methods, Xu et al. (2015) performed a genome-wide association study with the aim of identifying common genetic polymorphisms associated with overall and disease-free survival times in stage I-IV colorectal cancer patient cohorts [17]. This study did not identify associations reaching genome-wide significance levels. In addition, Phipps et al. (2016) investigated associations between genome-wide common genetic variants and survival outcomes in patients enrolled in six prospective cohort studies [51]. These authors also performed an analysis on a sub-group of their study cohort by focusing only on those patients who had already experienced metastasis at diagnosis (i.e. stage IV patients) and identified a set of SNPs in their pooled analysis that were significantly associated with overall survival times. In contrast to these studies, our study considered time-to-metastasis as the survival outcome, applied appropriate statistical methods due to the investigation of metastasis, and focused on patients with stage I-III MSI-L/MSS tumors only. Thus, this study differs from both previous studies and brings a new depth into colorectal cancer research in terms of its design and significant findings.

A large strength of this study is the comprehensive study design. We applied appropriate methods of analysis based on the endpoint of choice and the characteristics of the patient cohort subgroups we considered rather than applying the widely used Cox proportional hazards model only. In addition, by concentrating our efforts on a sub-cohort determined by the MSI tumor status and the tumor stage, we obtained a more homogeneous study cohort with an undifferentiated survival pattern (Fig. 1). This enabled us to reduce the genetic and phenotypic variability in the cohort to identify potential prognostic biomarkers. We also focused our efforts on Caucasian colorectal cancer patients exclusively, so these results may apply only to the Caucasian population. This intricate study design allowed for a more powerful analysis although we had a moderate number of patients. We also applied four genetic models to ensure a complete and informative investigation. However, some of the genetic models created genotype frequencies that were rare in this patient cohort, which resulted in a loss of power in the analysis of these genotype categories. Furthermore, while we included low-frequency SNPs (MAF 1–5%) in this study, no associations with such SNPs reached genome-wide significance (Table 4). Finally, it is important to note that in this study, we proposed and applied a framework for conducting a genome-wide association study of time-to-metastasis in curable cancer types. The study design and statistical methods utilized in this study are pertinent to any cancer type that has a large proportion of long-term metastasis-free survivors. This is significant, since advances in medical research are creating more patient cohorts with such a characteristic. Consequently, this study not only identified potential biomarkers for early metastasis in colorectal cancer patients, but also demonstrated an advanced and informative analysis approach to potentially enrich prognostic research in other cancer types.

## Conclusions

This is the first study to investigate genetic associations with time-to-metastasis in Caucasian colorectal cancer patients using such a large genetic data set and the first study where a mixture cure model was used for a high dimensional genetic data analysis. More importantly, for the first time, significant associations between genome-wide SNP genotype data and time-to-metastasis in Caucasian colorectal cancer patients were detected. The identified genetic variations represent a novel set of SNPs and genes that may have biological roles in colorectal cancer progression and metastasis in these patients. Once replicated, these results could aid in providing a means to distinguish colorectal cancer patients who are at an increased risk of early metastasis, which could be valuable in the clinical care of these patients as well as contribute to individualized therapies.

## Additional file

Additional file 1: **Table S1.** Results from the stepwise variable selection method using multivariable mixture cure model and Cox proportional hazards regression model to determine the final significant baseline characteristics. **Table S2.** Demographic and clinicopathologic characteristics of the patient cohort and *larger NFCCR cohort. **Table S3.** Genotypes significantly associated with time-to-metastasis identified in the univariable analysis using the mixture cure model. **Figure S1.** Conditional survival functions for the nine SNPs identified in the univariable analysis using the mixture cure model. **Table S4.** Results for all significant SNPs in the univariable Cox proportional hazards analysis and subsequent multivariable results. **Table S5.** Most significant associations with the long-term risk of metastasis estimated in the univariable mixture cure model. **Figure S2.** Kaplan-Meier survival function estimates for SNPs with the strongest association to long-term risk of metastasis in the mixture cure model. **Figure S3.** Known and hypothesized links between the intergenic SNPs, nearby genes, and the risk of metastasis. **Figure S4**. Kaplan-Meier survival function estimates for the nine SNPs significantly associated with time-to-metastasis after adjusting for significant baseline characteristics in the Cox proportional hazards regression model. (DOCX 1143 kb)

## Abbreviations

5-FU: 5-fluorouracil
CI: Confidence interval
DNA: Deoxyribonucleic acid
HR: Hazard ratio
HREB: Health Research Ethics Board
LD: Linkage disequilibrium
MAF: Minor allele frequency
MSI: Microsatellite instability
MSI-H: Microsatellite instability-high
MSI-L: Microsatellite instability-low
MSS: Microsatellite stable
ND: No data
NFCCR: Newfoundland and Labrador Colorectal Cancer Registry
OR: Odds ratio
RNA: Ribonucleic acid
SNP: Single nucleotide polymorphism
UCSC: University of California, Santa Cruz
USA: United States of America
