# Correction to: A genome-wide association study identifies single nucleotide polymorphisms associated with time-to-metastasis in colorectal cancer

**DOI:** 10.1186/s12885-019-5672-7

**Published:** 2019-05-10

**Authors:** Michelle E. Penney, Patrick S. Parfrey, Sevtap Savas, Yildiz E. Yilmaz

**Affiliations:** 1 Discipline of Genetics, Faculty of Medicine, Memorial University of Newfoundland, St. John’s, Canada; 2 Discipline of Medicine, Faculty of Medicine, Memorial University of Newfoundland, St. John’s, Canada; 3 Discipline of Oncology, Faculty of Medicine, Memorial University of Newfoundland, St. John’s, Canada; 4 Department of Mathematics and Statistics, Faculty of Science, Memorial University of Newfoundland, St. John’s, Canada

**Correction to: Penney et al. BMC Cancer (2019) 19:133**

**https://doi.org/10.1186/s12885-019-5346-5**

Following publication of the original article [1], the authors reported that Fig. 3 was mistakenly replaced by Fig. 4. The correct Fig. 3 is given below:

**Fig. 3:**
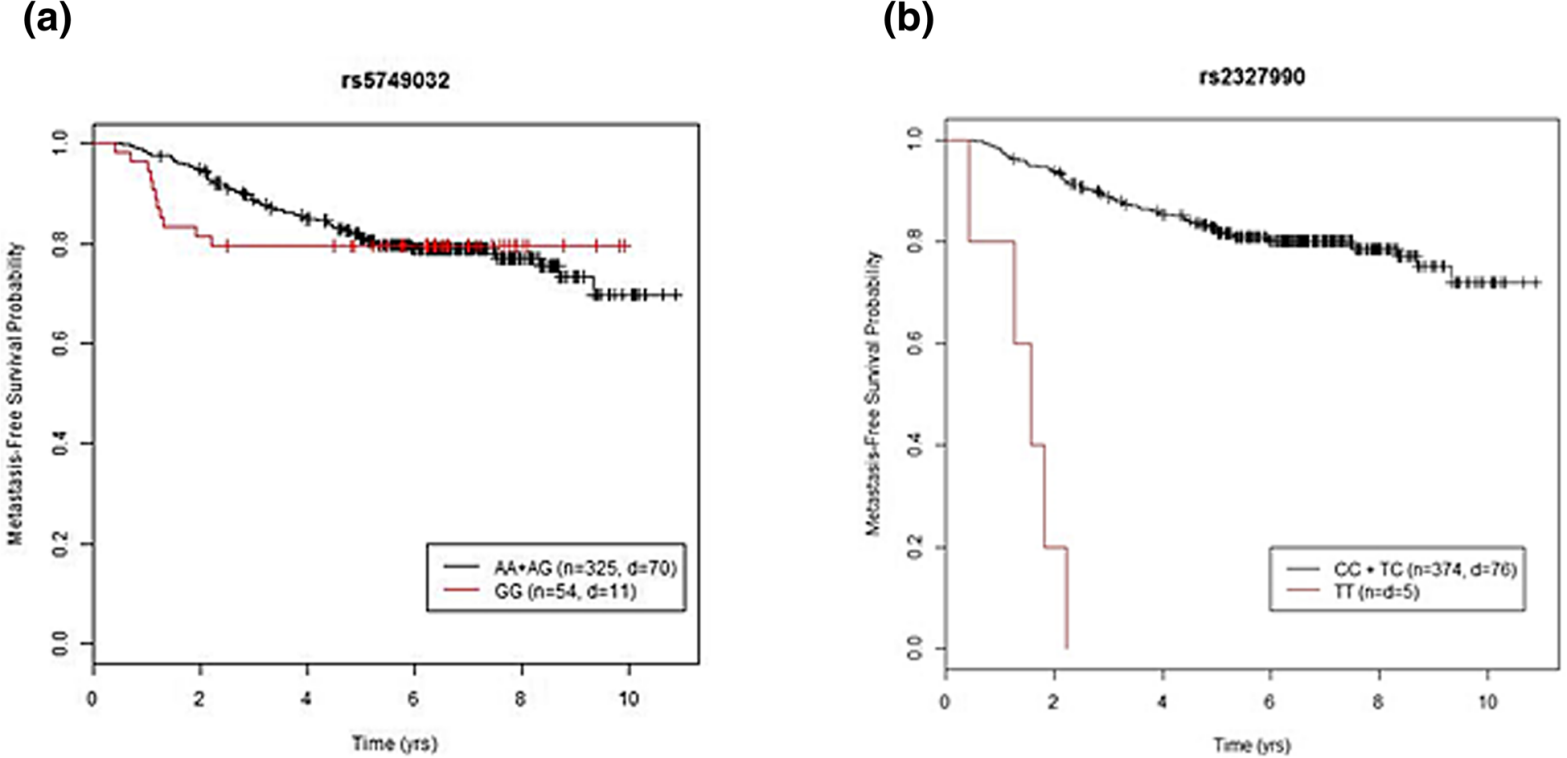
Kaplan-Meier survival function for the most significant SNPs in the multivariable analysis under the (**a**) mixture cure model and (**b**) Cox proportional hazards regression model. n: number of patients in that genotype category; d: number of metastasis in that genotype category. **a** rs5749032 was the only SNP maintaining genome-wide significance after the multivariable analysis using the mixture cure model. In the rs5749032 GG genotype subgroup, the clear plateau at approximately 80% metastasis-free survival probability indicates the existence of a large proportion of long-term metastasis-free survivors. **b** In the rs2327990 TT genotype subgroup, all the patients experienced metastasis within approximately the first two years. Therefore, a standard survival analysis method is appropriate
