# Supplementary material for: A genome-wide association study identifies single nucleotide polymorphisms associated with time-to-metastasis in colorectal cancer
Source: BMC Cancer. 2019 Feb 9;19:133. doi: 10.1186/s12885-019-5346-5 (PMC6368959; doi:10.1186/s12885-019-5346-5)
Supplement: Supplementary file 1 — Table S1. Results from the stepwise variable selection method using multivariable mixture cure model and Cox proportional hazards regression model to determine the final significant baseline characteristics. Table S2. Demographic and clinicopathologic characteristics of the patient cohort and *larger NFCCR cohort. Table S3. Genotypes significantly associated with time-to-metastasis identified in the univariable analysis using the mixture cure model. Figure S1. Conditional survival functions for the nine SNPs identified in the univariable analysis using the mixture cure model. Table S4. Results for all significant SNPs in the univariable Cox proportional hazards analysis and subsequent multivariable results. Table S5. Most significant associations with the long-term risk of metastasis estimated in the univariable mixture cure model. Figure S2. Kaplan-Meier survival function estimates for SNPs with the strongest association to long-term risk of metastasis in the mixture cure model. Figure S3. Known and hypothesized links between the intergenic SNPs, nearby genes, and the risk of metastasis. Figure S4. Kaplan-Meier survival function estimates for the nine SNPs significantly associated with time-to-metastasis after adjusting for significant baseline characteristics in the Cox proportional hazards regression model. (DOCX 1143 kb) [file 12885_2019_5346_MOESM1_ESM.docx]

**Additional File**

**A Genome-wide Association Study Identifies Single Nucleotide Polymorphisms Associated with Time-to-Metastasis in Colorectal Cancer**

**Michelle E. Penney, Patrick S. Parfrey, Sevtap Savas, Yildiz E. Yilmaz**

**Supplementary Tables and Figures**

Supplementary Table 1. Results from the stepwise variable selection method using multivariable mixture cure model and Cox proportional hazards regression model to determine the final significant baseline characteristics

Supplementary Table 2. Demographic and clinicopathologic characteristics of the patient cohort and *larger NFCCR cohort

Supplementary Table 3. Genotypes significantly associated with time-to-metastasis identified in the univariable analysis using the mixture cure model

Supplementary Figure 1. Conditional survival functions for the nine SNPs identified in the univariable analysis using the mixture cure model

Supplementary Table 4. Results for all significant SNPs in the univariable Cox proportional hazards analysis and subsequent multivariable results

Supplementary Table 5. Most significant associations with the long-term risk of metastasis estimated in the univariable mixture cure model

Supplementary Figure 2. Kaplan-Meier survival function estimates for SNPs with the strongest association to long-term risk of metastasis in the mixture cure model.

Supplementary Figure 3. Known and hypothesized links between the intergenic SNPs, nearby genes, and the risk of metastasis

Supplementary Figure 4. Kaplan-Meier survival function estimates for the nine SNPs significantly associated with time-to-metastasis after adjusting for significant baseline characteristics in the Cox proportional hazards regression model

**Supplementary Data References**

**Supplementary Tables and Figures**

**Supplementary Table 1.** Results from the stepwise variable selection method using multivariable mixture cure model and Cox proportional hazards regression model to determine the final significant baseline characteristics

| **Mixture cure model** |  | ***Logistic regression model for metastasis probability*** | | |  | ***Proportional hazards model for time-to-metastasis*** | | |
| --- | --- | --- | --- | --- | --- | --- | --- | --- |
|  |  |  |  |  |  |  |  |  |
|  | **Variable (*a* vs. *b*)** | **OR** | **95% CI** | **p-value** |  | **HR** | **95% CI** | **p-value** |
|  | Location (rectum vs. colon) | 9.11 | 1.05-78.82 | 0.0447 |  | 0.23 | 0.09-0.58 | 0.0018 |
|  | 5-FU treatment status (given vs. not given/unknown) | 7.15 | 1.37-37.26 | 0.0195 |  | 0.21 | 0.06-0.69 | 0.0103 |
|  | Stage II (vs. Stage I) | 1.25 | 0.12-13.47 | 0.8529 |  | 3.45 | 0.34-35.37 | 0.2964 |
|  | Stage III (vs. Stage I) | 0.45 | 0.02-8.75 | 0.5990 |  | 14.22 | 1.27-159.49 | 0.0313 |
| **Cox proportional hazards regression model** |  | ***Cox PH model for time-to-metastasis*** | | | | | | |
|  | **Variable (*a* vs. *b*)** | **HR** | | **95% CI** | | | **p-value** | |
|  | 5-FU treatment status (given vs. not given/unknown) | 1.37 | | 0.64 - 2.91 | | | 0.4078 | |
|  | Stage II (vs. Stage I) | 1.92 | | 0.71 - 5.17 | | | 0.1986 | |
|  | Stage III (vs. Stage I) | 3.10 | | 1.04 - 9.24 | | | 0.0424 | |
|  | Location (rectum vs. colon) | 1.76 | | 1.06 - 2.92 | | | 0.0278 | |
|  | *BRAF* V600E mutation (present vs. absent) | 2.83 | | 1.30 - 6.16 | | | 0.0085 | |

OR: odds ratio for metastasis (i.e. probability of being in the susceptible group). OR compares metastasis proportion in subgroup *a* with that in subgroup *b*. HR: hazard ratio for time to metastasis among susceptible patients. HR compares metastasis rate in subgroup *a* with that in subgroup *b* among those who are susceptible to metastasis. CI: confidence interval; 5-FU: 5-fluorouracil.

**Supplementary Table 2.** Demographic and clinicopathologic characteristics of the patient cohort and *larger NFCCR cohort

| **Variable** | | **Number of Patients in NFCCR Cohort (n=493)*** | **% Total** | **Number of Patients in Sample Cohort (n=379)** | **% Total** |
| --- | --- | --- | --- | --- | --- |
| Sex | Female | 181 | 36.7% | 139 | 36.7% |
|  | Male | 312 | 63.3% | 240 | 63.3% |
| Age | ≤60 | 191 | 38.7% | 157 | 41.4% |
|  | 60-70 | 207 | 42.0% | 154 | 40.6% |
|  | >70 | 95 | 19.3% | 68 | 17.9% |
| Familial risk | Low | 248 | 50.3% | 196 | 51.7% |
|  | Intermediate/high | 231 | 46.9% | 183 | 48.3% |
|  | Unknown | 14 | 2.8% | 0 | 0% |
| 5-FU based treatment | 5-FU treated | 268 | 54.4% | 214 | 56.5% |
|  | other/no chemo | 216 | 43.8% | 159 | 42.0% |
|  | Unknown | 9 | 1.8% | 6 | 1.6% |
| Stage | I | 92 | 18.7% | 81 | 21.4% |
|  | II | 201 | 40.8% | 158 | 41.7% |
|  | III | 200 | 40.6% | 140 | 36.9% |
| Location | Colon | 309 | 62.7% | 233 | 61.5% |
|  | Rectum | 184 | 37.3% | 146 | 38.5% |
| Histology | Non-mucinous | 441 | 89.5% | 343 | 90.5% |
|  | Mucinous | 52 | 10.5% | 36 | 9.5% |
| Vascular invasion | Absence | 299 | 60.6% | 242 | 63.9% |
|  | Presence | 161 | 32.7% | 111 | 29.3% |
|  | Unknown | 33 | 6.7% | 26 | 6.9% |
| Lymphatic invasion | Absence | 293 | 59.4% | 237 | 62.5% |
|  | Presence | 163 | 33.1% | 116 | 30.6% |
|  | Unknown | 37 | 7.5% | 26 | 6.9% |
| *BRAF V600E* mutation | Absence | 426 | 86.4% | 333 | 87.9% |
|  | Presence | 35 | 7.1% | 19 | 5.0% |
|  | Unknown | 32 | 6.5% | 27 | 7.1% |

5-FU: 5-fluorouracil. *NFCCR included 750 consenting patients diagnosed with colorectal cancer between 1999 and 2003 ^1,2^. From this set of patients, only the patients with MSI-L/MSS tumors and Stage I-III patients are shown in this table.

**Supplementary Table 3.** Genotypes significantly associated with time-to-metastasis identified in the univariable analysis using the mixture cure model

|  | | | ***Logistic regression model for metastasis probability*** | | |  | ***Proportional hazards model for time-to-metastasis*** | | |
| --- | --- | --- | --- | --- | --- | --- | --- | --- | --- |
| **Genomic Location** | **Genetic Model** | **rs Number (genotypes *a* vs. *b*)** | **OR** | **95% CI** | **p-value** |  | **HR** | **95% CI** | **p-value** |
| 22:17793969 | Recessive | rs5749032  (GG vs. AA + AG) | 0.73 | 0.35 - 1.53 | 0.400 |  | 9.55 | 4.44 - 20.55 | 7.70×10^-9^ |
| 17:77361176 | Co-Dominant | rs12949587  (CT vs. CC) | 0.61 | 0.31 - 1.20 | 0.151 |  | 7.92 | 3.88 - 16.16 | 1.29×10^-8^ |
| 20:15111138 | Co-Dominant | rs6110524  (AG vs. GG) | 0.86 | 0.44 - 1.70 | 0.665 |  | 7.56 | 3.75 - 15.27 | 1.66×10^-8^ |
| 7:33913404 | Recessive | rs3815652  (TT vs. CC + CT) | 1.38 | 0.46 - 4.15 | 0.566 |  | 20.75 | 7.20 - 59.80 | 1.96×10^-8^ |
| 14:100691178 | Recessive | rs756055  (CC vs. TT + TC) | 0.44 | 0.18 - 1.03 | 0.058 |  | 13.39 | 5.37 - 33.43 | 2.70×10^-8^ |
| 14:100730920 | Recessive | rs7153665  (AA vs. GG + AG) | 0.44 | 0.18 - 1.04 | 0.058 |  | 13.39 | 5.37 - 33.44 | 2.70×10^-8^ |
| 11:100430053 | Recessive | rs4754687  (AA vs. CC + CA) | 0.60 | 0.25 - 1.44 | 0.255 |  | 13.33 | 5.34 - 33.28 | 2.90×10^-8^ |
| 5:155345221 | Dominant | rs2163746  (CT + CC vs. TT) | 0.60 | 0.31 - 1.15 | 0.124 |  | 6.45 | 3.29 - 12.63 | 5.40×10^-8^ |
| 5:155361116 | Dominant | rs17053011  (TG + TT vs. GG) | 0.60 | 0.31 - 1.16 | 0.124 |  | 6.45 | 3.29 - 12.64 | 5.40×10^-8^ |

OR: odds ratio for metastasis (i.e. probability of being in the susceptible group). OR compares metastasis proportion in subgroup *a* with that in subgroup *b*. HR: hazard ratio for time to metastasis among susceptible patients. HR compares metastasis rate in subgroup *a* with that in subgroup *b* among those who are susceptible to metastasis. CI: confidence interval.

**Supplementary Figure 1.** Conditional survival functions for the nine SNPs identified in the univariable analysis using the mixture cure model


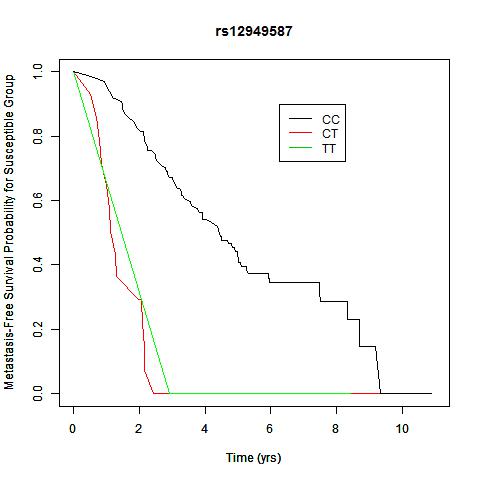

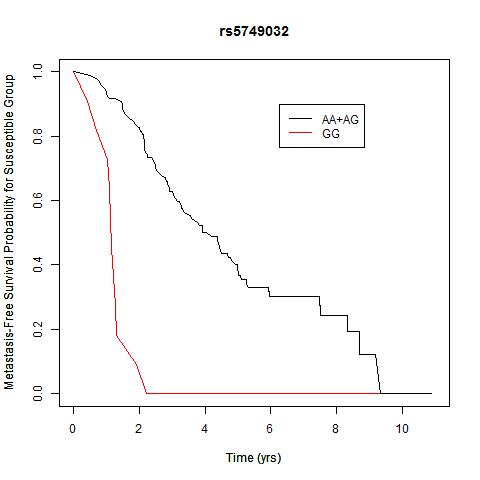

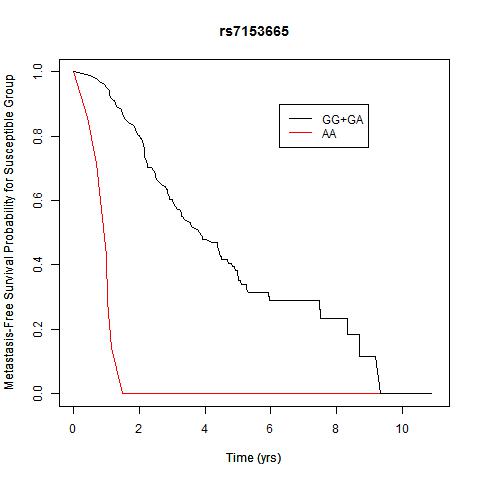

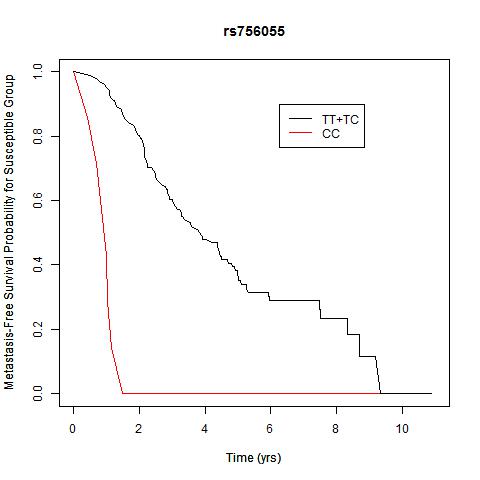

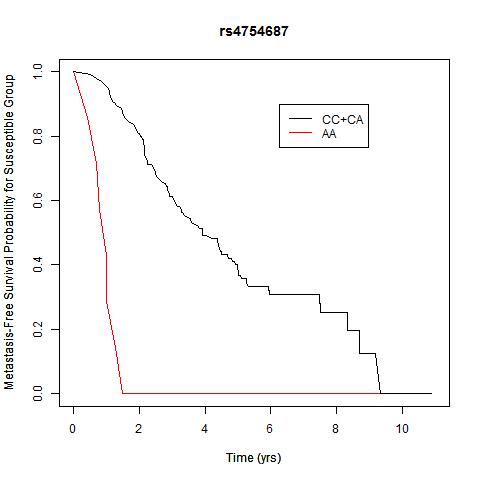

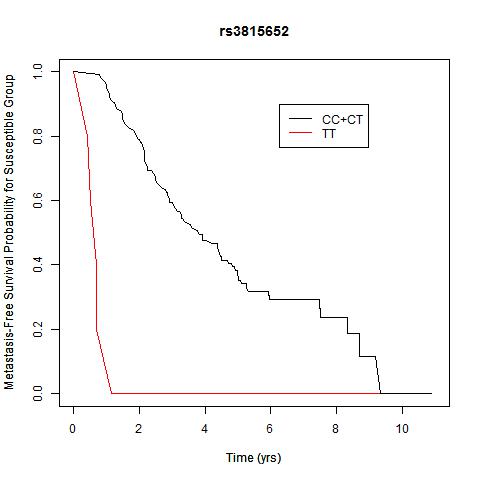

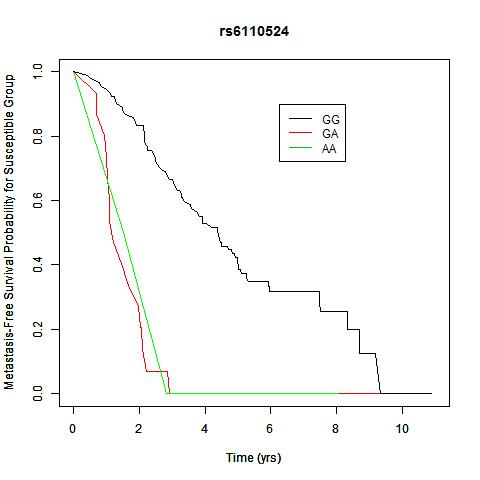

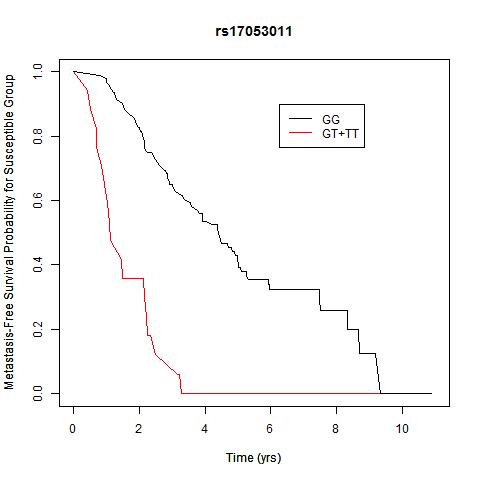

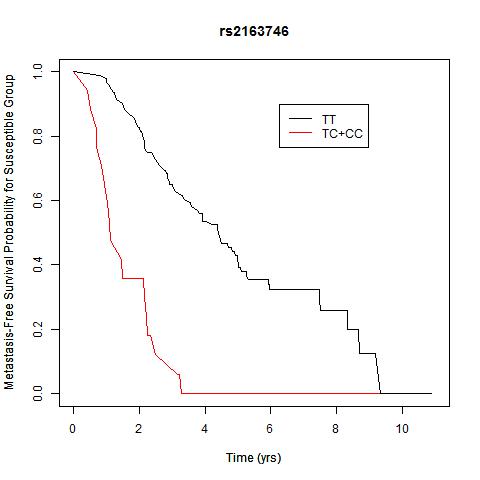


Under the assumptions of the mixture cure model, the population is viewed as a mixture of susceptible and non-susceptible individuals to metastasis, where susceptible refers to patients who will experience metastasis and non-susceptible individuals are long-term metastasis-free survivors who are viewed as (statistically) cured. For example, Figure 3a shows the Kaplan-Meier estimate of the survival curves of time-to-metastasis ($T$) for each genotype category of a specific SNP rs5749032 (i.e., Kaplan-Meier estimates of the survival function $S(t|x) = Pr(T>t|x)$ where $x$ denotes the covariate (genotype category of the corresponding polymorphism)). On the other hand, the plots in this figure show the estimated conditional survival curves for the susceptible group under each $x$ level (i.e., Kaplan-Meier estimates of $S_{0}(t|x) = Pr(T > t | susceptible group, x)$ which is the probability that the susceptible person will survive beyond a specified time $t$ without metastasis). Hence, in Figure 3a, the probability of survival is for the population under consideration including both susceptible and non-susceptible individuals, but the plots in this figure are for the survival function of time-to-metastasis in the group of susceptible individuals. The conditional survival curves for the susceptible group are obtained from the mixture cure model $S(t|x) = Pr(T>t|x) = p(x)+(1-p(x))S_{0}(t|x)$ where $p(x)$ denotes the probability of being long-term metastasis-free survivor and thus $1-p(x)$ is the probability of being susceptible to metastasis. Hence, the curves in this figure were obtained by plugging the Kaplan-Meier estimates of $S(t|x)$ and $p(x)$ in $S_{0}(t|x) = (S(t|x)-p(x))/(1-p(x))$.

**Supplementary Table 4.** Results for all significant SNPs in the univariable Cox proportional hazards analysis and subsequent multivariable results

| **Genomic Location** | **rs Number (Genotype)** | **Univariable** | | |  | **Multivariable** | | |
| --- | --- | --- | --- | --- | --- | --- | --- | --- |
|  |  | **HR** | **95% CI** | **p-value** |  | **HR** | **95% CI** | **p-value** |
| 20:16189263 | rs2327990 (TT) | 21.97 | 8.42 - 57.33 | 2.74×10^-10^ |  | 22.58 | 8.32 - 61.31 | 9.59×10^-10^ |
| 3:134513356 | rs11918092 (CC) | 216.98 | 35.64 - 1321.13 | 5.32×10^-9^ |  | 535.33 | 63.20 - 4534.30 | 8.23×10^-9^ |
| 3:134515336 | rs3732568 (AA) | 216.98 | 35.64 - 1321.13 | 5.32×10^-9^ |  | 535.33 | 63.20 - 4534.30 | 8.23×10^-9^ |
| 3:59930672 | rs2366964 (CC) | 41.19 | 11.81 - 143.66 | 5.40×10^-9^ |  | 56.53 | 14.98 - 213.26 | 2.59×10^-9^ |
| 2:175205513 | rs7582977 (CC) | 134.32 | 25.76 - 700.33 | 6.02×10^-9^ |  | 82.61 | 14.50 - 470.67 | 6.63×10^-7^ |
| 13:48118782 | rs9534678 (AA) | 133.60 | 25.62 - 696.59 | 6.26×10^-9^ |  | 83.96 | 14.71 - 479.13 | 6.17×10^-7^ |
| 2:86015121 | rs13402783 (GG) | 20.91 | 7.47 - 58.50 | 6.94×10^-9^ |  | 13.03 | 4.50 - 37.78 | 2.25×10^-6^ |
| 2:86013029 | rs13386681 (TT) | 20.79 | 7.47 - 58.50 | 7.40×10^-9^ |  | 12.86 | 4.43 - 37.27 | 2.57×10^-6^ |
| 2:6769988 | rs1563948 (AA) | 34.43 | 10.35 - 114.58 | 7.97×10^-9^ |  | 33.97 | 9.57 - 120.54 | 4.87×10^-8^ |
| 2:6773920 | rs11692570 (TT) | 34.43 | 10.35 - 114.58 | 7.97×10^-9^ |  | 33.97 | 9.57 - 120.54 | 4.87×10^-8^ |
| 2:6777992 | rs2219613 (TT) | 34.43 | 10.35 - 114.58 | 7.97×10^-9^ |  | 33.97 | 9.57 - 120.54 | 4.87×10^-8^ |
| 2:6779277 | rs11694697 (TT) | 34.43 | 10.35 - 114.58 | 7.97×10^-9^ |  | 33.97 | 9.57 - 120.54 | 4.87×10^-8^ |
| 5:148172928 | rs9285673 (CC) | 36.70 | 10.53 - 127.95 | 1.56×10^-8^ |  | 19.47 | 5.41 - 70.13 | 5.60×10^-6^ |
| 15:89420974 | rs17201864 (TT) | 19.06 | 6.86 - 52.98 | 1.60×10^-8^ |  | 11.01 | 3.76 - 32.24 | 1.20×10^-5^ |
| 9:119519588 | rs1372330 (AA) | 36.51 | 10.47 - 127.34 | 1.67×10^-8^ |  | 27.15 | 7.66 - 96.27 | 3.20×10^-7^ |
| 6:91187510 | rs1145724 (GG) | 30.76 | 9.27 - 102.03 | 2.14×10^-8^ |  | 36.43 | 10.21 - 129.93 | 3.00×10^-8^ |
| 4:53893156 | rs17082301 (AA) | 129.98 | 23.29 - 725.52 | 2.89×10^-8^ |  | 81.63 | 8.85 - 753.27 | 0.0001 |
| 1:190131750 | rs10920654 (TT) | 76.85 | 16.51 - 357.84 | 3.16×10^-8^ |  | 32.48 | 5.86 - 180.04 | 6.78×10^-5^ |
| 10:98422896 | rs1023741 (CC) | 18.64 | 6.57 - 52.84 | 3.76×10^-8^ |  | 17.77 | 5.07 - 62.25 | 6.87×10^-6^ |
| 4:14296300 | rs1426107 (AA) | 28.08 | 8.53 - 92.45 | 4.13×10^-8^ |  | 14.10 | 2.70 - 73.53 | 0.0017 |
| 18:40691675 | rs3861289 (AA) | 19.16 | 6.62 - 55.40 | 5.04×10^-8^ |  | 8.73 | 2.58 - 29.54 | 0.0005 |
| 17:7396267 | rs4265880 (AA) | 98.28 | 18.72 - 515.88 | 5.86×10^-8^ |  | 64.74 | 11.27 - 371.86 | 2.93×10^-6^ |
| 17:7397043 | rs4239258 (TT) | 98.28 | 18.72 - 515.88 | 5.86×10^-8^ |  | 64.74 | 11.27 - 371.86 | 2.93×10^-6^ |
| 17:7404991 | rs2228130 (TT) | 98.28 | 18.72 - 515.88 | 5.86×10^-8^ |  | 64.74 | 11.27 - 371.86 | 2.93×10^-6^ |
| 17:7418109 | rs9989479 (AA) | 98.28 | 18.72 - 515.88 | 5.86×10^-8^ |  | 64.74 | 11.27 - 371.86 | 2.93×10^-6^ |

HR: hazard ratio for time to metastasis among susceptible patients. CI: confidence interval; 5-FU: 5-fluorouracil

**Supplementary Table 5.** Most significant associations with the long-term risk of metastasis estimated in the univariable mixture cure model

| **Genomic location** | **rs number (genotypes *a* vs. *b*)** | **Genetic model** | **Genotype freq.** | **MAF** | **Type of variant*** | ***Logistic regression model for metastasis probability*** | | |  | ***Proportional hazards model for time-to-metastasis*** | | |
| --- | --- | --- | --- | --- | --- | --- | --- | --- | --- | --- | --- | --- |
|  |  |  |  |  |  | **OR** | **95% CI** | **p-value** |  | **HR** | **95% CI** | **p-value** |
| 8:65783019 | rs6985116 (CC vs. TT) | Co-Dominant | 23% | 48% | Intergenic | 0.07 | 0.02-0.24 | 1.82×10^-5^ |  | 2.61 | 0.68-10.07 | 0.162 |
| 8:5438981 | rs17354999 (AG & AA vs. GG) | Additive | 66% | 44% | Intergenic | 2.93 | 1.79-4.79 | 1.98×10^-5^ |  | 0.62 | 0.38-1.00 | 0.052 |
| 5:105924416 | rs10080115 (CT vs. TT) | Co-Dominant | 39% | 27% | Intergenic | 0.24 | 0.13-0.47 | 2.03×10^-5^ |  | 2.32 | 1.15-4.71 | 0.020 |
| 22:47701711 | rs4823630 (TC vs. CC) | Co-Dominant | 46% | 34% | Intergenic | 0.24 | 0.12-0.47 | 2.58×10^-5^ |  | 1.33 | 0.60-2.93 | 0.484 |
| 8:5437805 | rs1468386 (AA vs. GG) | Co-Dominant | 25% | 49% | Intergenic | 7.41 | 2.87-19.17 | 3.61×10^-5^ |  | 0.64 | 0.21-1.95 | 0.430 |

*based on Ensembl database ^3^.

OR: odds ratio for metastasis comparing odds of metastasis in subgroup *a* with that in subgroup *b*. HR: hazard ratio comparing metastasis rate in subgroup *a* with that in subgroup *b* among those who are susceptible to metastasis. MAF: minor allele frequency calculated from patient cohort analyzed; Genotype freq.: frequency of genotype *a* calculated from the patient cohort; CI: confidence interval.

These SNPs were all located in intergenic regions and have no known regulatory consequences according to the RegulomeDB database ^4^. The results contained odds ratio estimates different than 1 (p<3.7×10^-5^), indicating that these SNPs could be differentiators for being long-term metastasis free survivors, but the associations did not reach the conservative Bonferroni-corrected significance level. This could be indicative of a lack of power due to the small number of patients who experienced metastasis. Consequently, these SNPs should be investigated in a larger cohort.

**Supplementary Figure 2.** Kaplan-Meier survival function estimates for SNPs with the strongest association to long-term risk of metastasis in the mixture cure model.


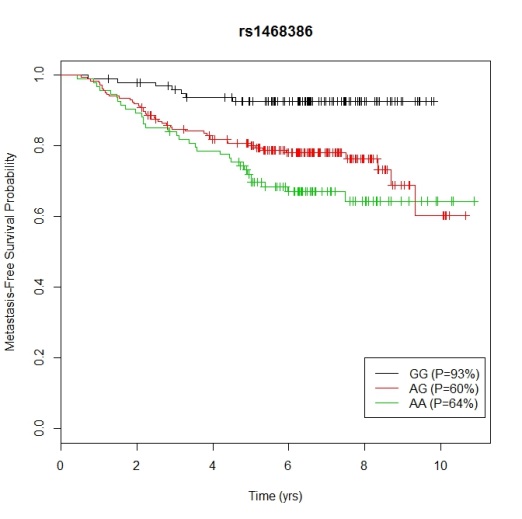

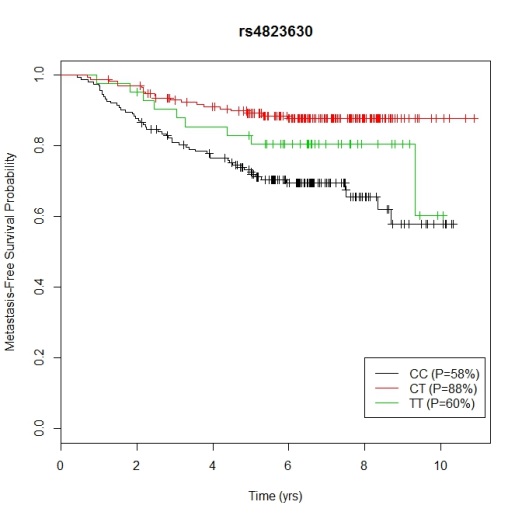

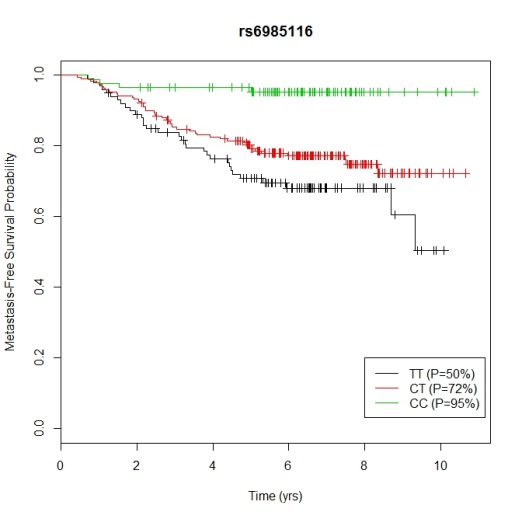

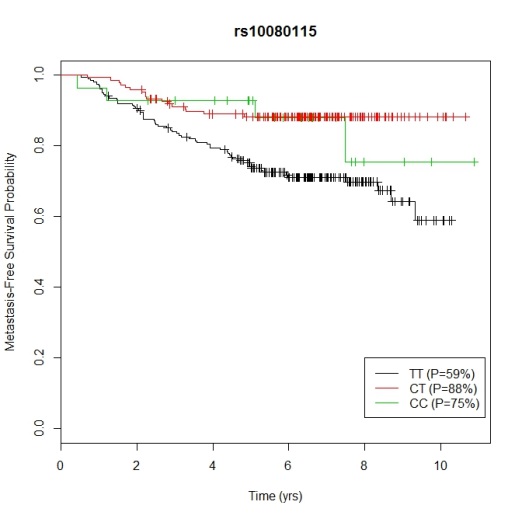

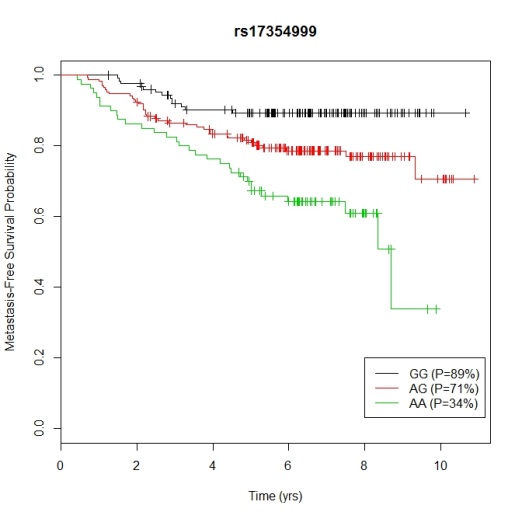


**Supplementary Figure 3.** Known and hypothesized links between the intergenic SNPs, nearby genes, and the risk of metastasis


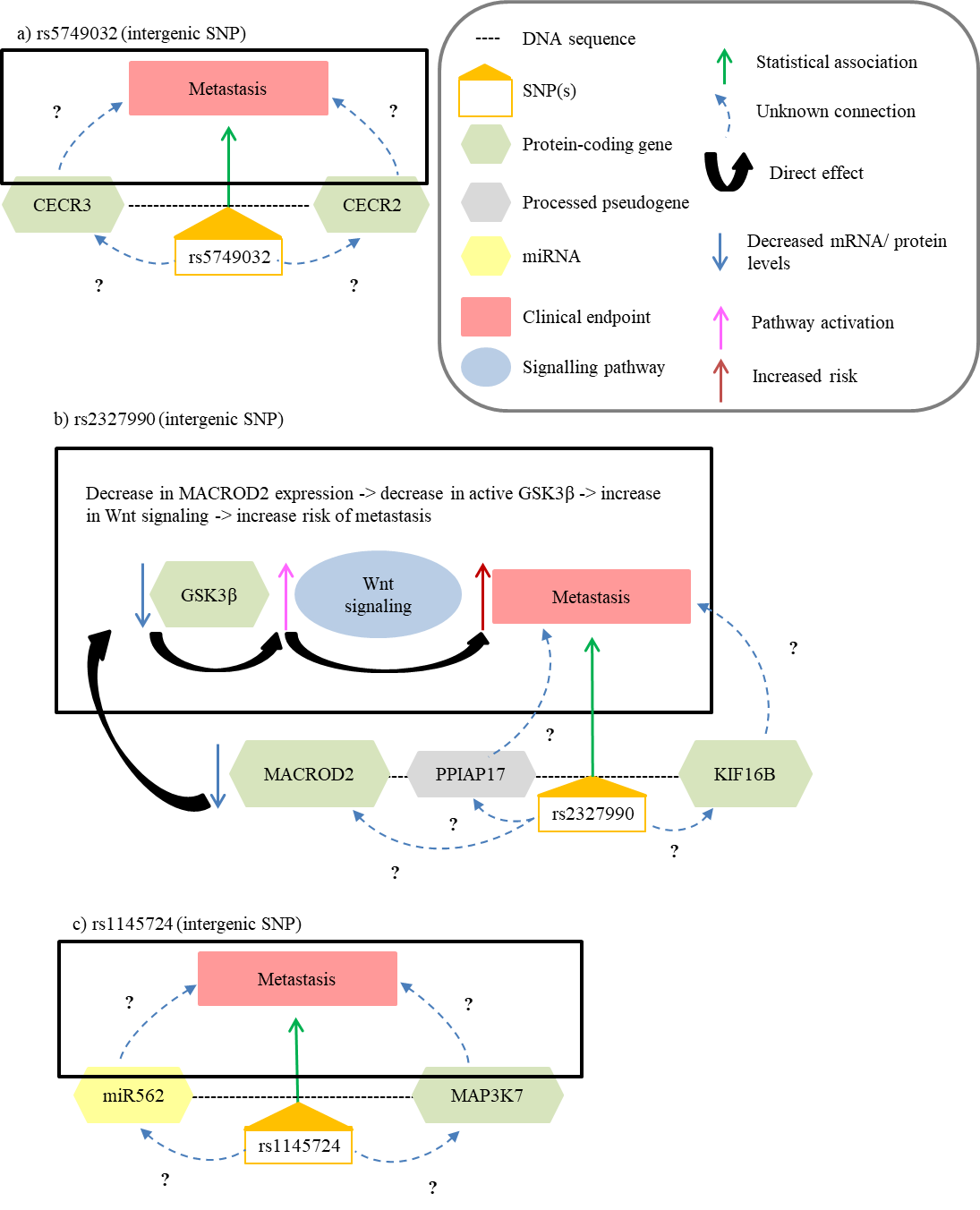


All SNPs except rs1145724, are discussed in Discussion section of the manuscript. The intergenic SNP rs1145724 was identified by the Cox PH model as significantly associated with time to metastasis. According to UCSC genome browser ^5^ this SNP is flanked by a miRNA, *miR562*, and a mitogen-activated protein kinase gene, *MAP3K7*. There is no scientific literature linking *miR562* to colorectal cancer. MAP3K7, on the other hand, has been shown to be linked to colorectal cancer in several studies ^6-8^. MAP3K7 (TAK1) mediates signal transduction in several pathways, including negative regulation of Wnt signaling ^9^. However, at the present time there is no known connection between this SNP, these genes, or colorectal cancer metastasis. It is also possible that the SNPs identified in this study may have long-distance regulatory functions.

**Supplementary Figure 4**. Kaplan-Meier survival function estimates for the nine SNPs significantly associated with time-to-metastasis after adjusting for significant baseline characteristics in the Cox proportional hazards regression model


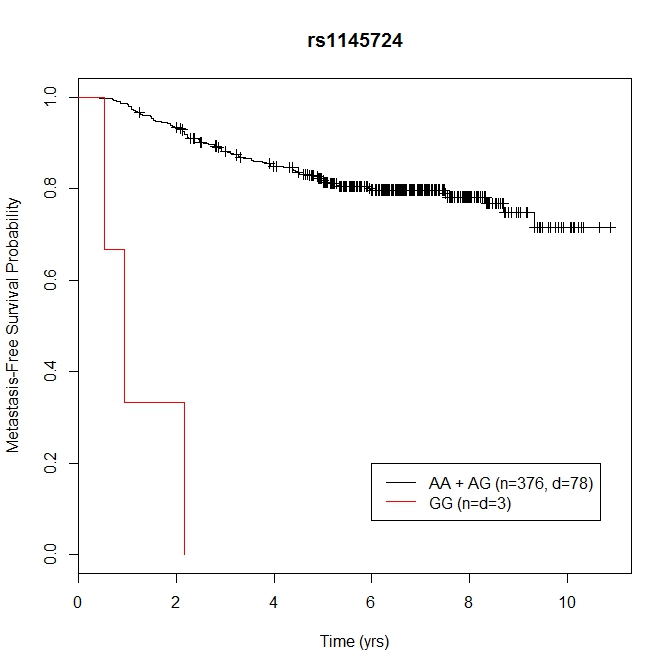

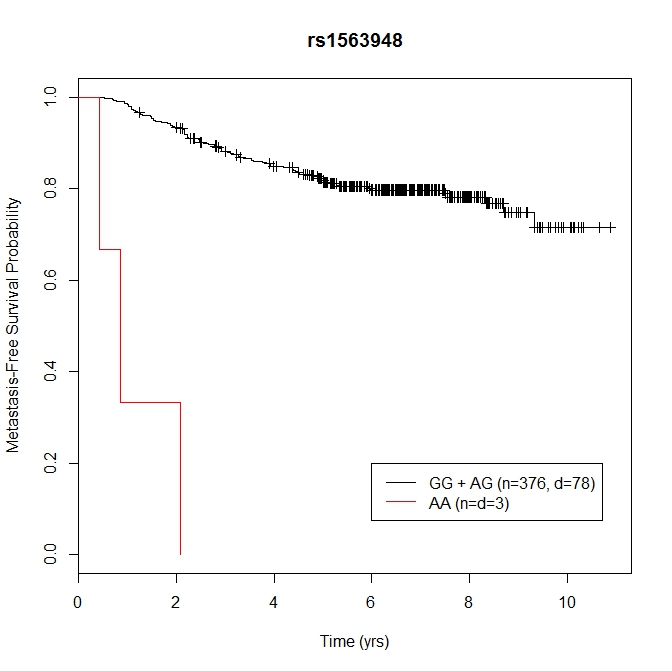

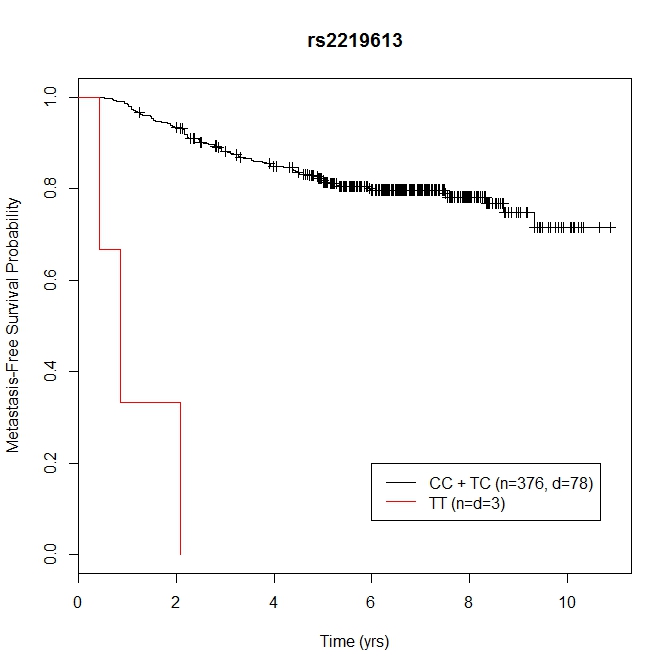


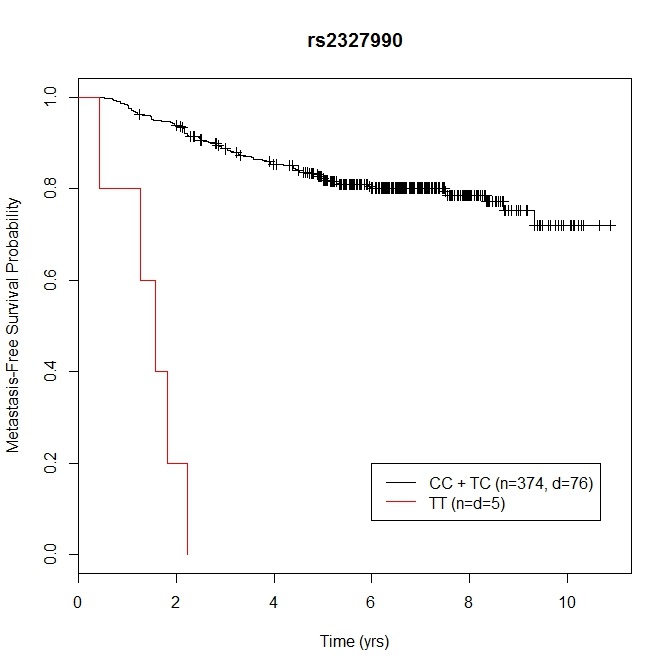

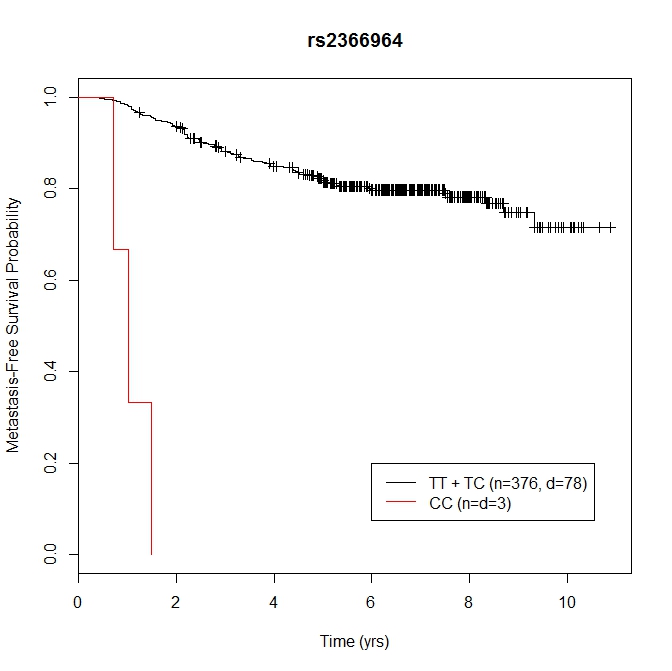

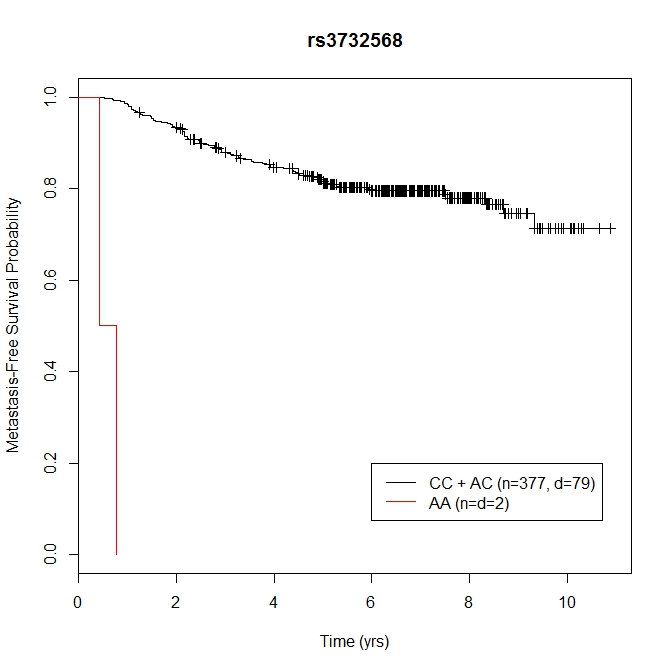


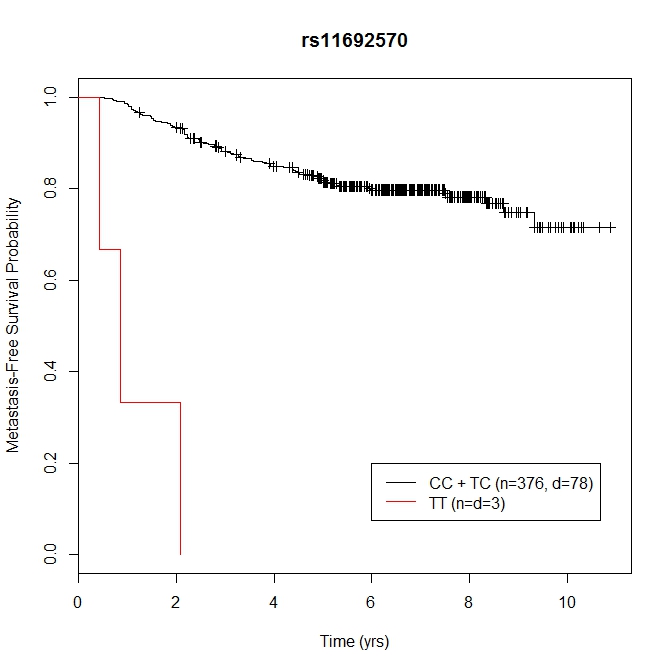

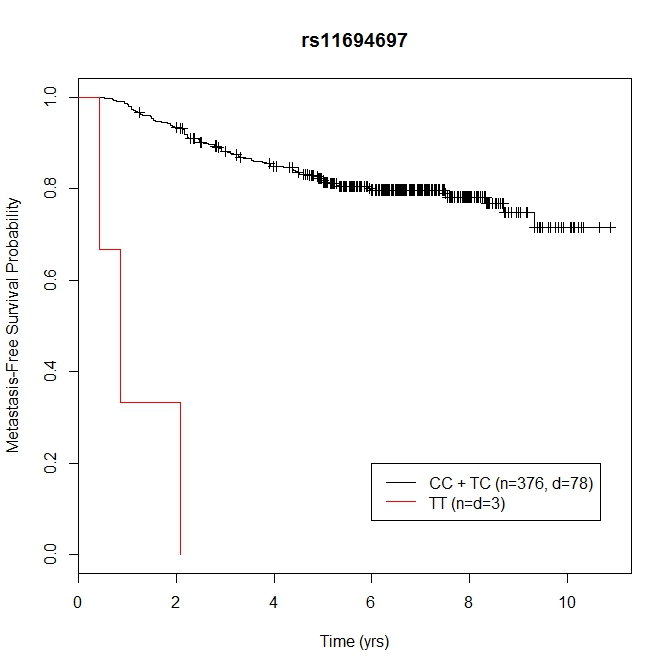

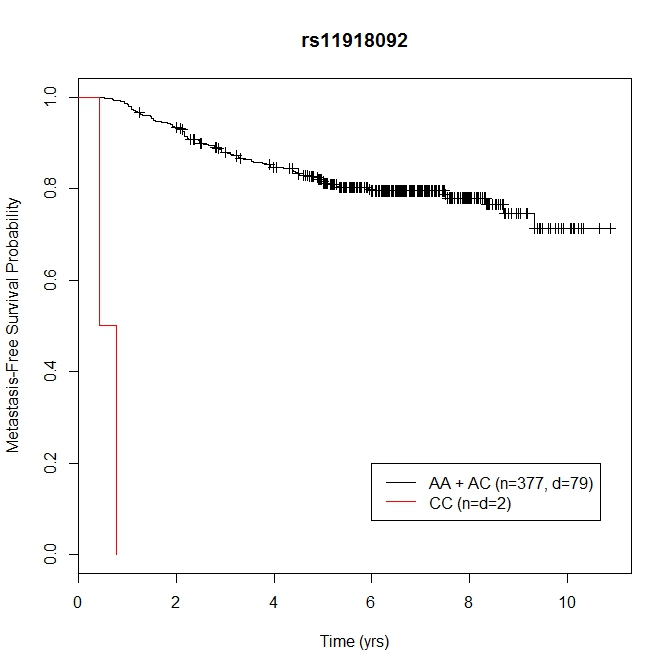


n=number of patients in that specific genotype category; d=number of patients in the genotype category who experience metastasis within the follow-up time.

**Supplementary Data References**

1. Wish TA, Hyde AJ, Parfrey PS, et al. Increased cancer predisposition in family members of colorectal cancer patients harboring the p.V600E BRAF mutation: A population-based study. *Cancer Epidemiol Biomarkers Prev*. 2010;19(7):1831-1839.

2. Woods MO, Younghusband HB, Parfrey PS, et al. The genetic basis of colorectal cancer in a population-based incident cohort with a high rate of familial disease. *Gut*. 2010;59(10):1369-1377.

3. Flicek P, Amode MR, Barrell D, et al. Ensembl 2014. *Nucleic Acids Res*. 2014;42(D1):D749-D755.

4. Boyle AP, Hong EL, Hariharan M, et al. Annotation of functional variation in personal genomes using RegulomeDB. *Genome Res*. 2012;22(9):1790-1797.

5. Kent WJ, Sugnet CW, Furey TS, et al. The human genome browser at UCSC. *Genome Res.* 2002;12(6):996-1006.

6. Slattery ML, Lundgreen A, Wolff RK. Dietary influence on MAPK-signaling pathways and risk of colon and rectal cancer. *Nutr Cancer*. 2013;65(5):729-738.

7. Takahashi H, Jin C, Rajabi H, et al. Muc1-c activates the tak1 inflammatory pathway in colon cancer. *Oncogene*. 2015;34(40):5187-5197.

8. Singh A, Sweeney MF, Yu M, et al. TAK1 (MAP3K7) inhibition promotes apoptosis in KRAS-dependent colon cancers. *Cell*. 2012;148(4):639-650.

9. Behrens J. Cross-regulation of the Wnt signalling pathway: A role of MAP kinases. *J Cell Sci*. 2000;113(6):911.
